# Spatial-temporal Bayesian accelerated failure time models for survival endpoints with applications to prostate cancer registry data

**DOI:** 10.1186/s12874-024-02201-w

**Published:** 2024-04-08

**Authors:** Ming Wang, Zheng Li, Jun Lu, Lijun Zhang, Yimei Li, Liangliang Zhang

**Affiliations:** 1 Department of Population and Quantitative Health Sciences, Case Western Reserve University, Cleveland, OH USA; 2 Novartis Pharmaceuticals, East Hanover, NJ USA; 3 Division of Epidemiology and Biostatistics, School of Public Health, University of Illinois Chicago, Chicago, IL USA; 4 Department of Biostatistics, Epidemiology, and Informatics, University of Pennsylvania, Philadelphia, PA USA

**Keywords:** Accelerated failure times, Bayesian inference, Monte Carlo Markov chain, Multivariate conditional autoregressive priors, Prostate cancer, Spatial-temporal modeling

## Abstract

Prostate cancer is the most common cancer after non-melanoma skin cancer and the second leading cause of cancer deaths in US men. Its incidence and mortality rates vary substantially across geographical regions and over time, with large disparities by race, geographic regions (i.e., Appalachia), among others. The widely used Cox proportional hazards model is usually not applicable in such scenarios owing to the violation of the proportional hazards assumption. In this paper, we fit Bayesian accelerated failure time models for the analysis of prostate cancer survival and take dependent spatial structures and temporal information into account by incorporating random effects with multivariate conditional autoregressive priors. In particular, we relax the proportional hazards assumption, consider flexible frailty structures in space and time, and also explore strategies for handling the temporal variable. The parameter estimation and inference are based on a Monte Carlo Markov chain technique under a Bayesian framework. The deviance information criterion is used to check goodness of fit and to select the best candidate model. Extensive simulations are performed to examine and compare the performances of models in different contexts. Finally, we illustrate our approach by using the 2004-2014 Pennsylvania Prostate Cancer Registry data to explore spatial-temporal heterogeneity in overall survival and identify significant risk factors.

**Supplementary Information:**

The online version contains supplementary material available at 10.1186/s12874-024-02201-w.

## Introduction

Prostate cancer (PC) is the most common cancer after non-melanoma skin cancer and the second leading cause of cancer deaths in US men, with 31,620 deaths estimated in 2019, a 7% increase compared with 2018 [1]. In recent years, PC care and outcomes have substantially improved, with a 5-year survival rate of up to 100% if the cancer is diagnosed at an early stage; however, these improvements are not equally shared across geographic regions, and elevated mortality has been observed among patients in some specific areas (i.e., rural or Appalachian regions) [2]. One important potential factor driving this geographic disparity is access to high-quality cancer care. Other disparities have also been explored, including black race, older age, and family history of PC [2]. Despite the high 5-year survival rate with early diagnosis, the diagnosis of PC is likely to be delayed owing to the aforementioned factors. Thus, improving PC survival outcomes is still crucial and challenging, and there is a need to better understand the spatial-temporal heterogeneity of PC and identify high-risk populations to enable more effective implementation of screening policies and intervention strategies.

To achieve these goals, existing population-based cancer registry data provide fruitful resources and platforms for analysis; however, there are several specific data issues: 1) substantially multi-modal risk factors with various data types; 2) spatial-temporal variation in cancer mortality, with adjacent neighborhoods or temporal cohorts more alike than those from distant regions or years owing to similar environmental and social factors [3–6]; and 3) the availability of individual-level data for analysis. In this article, we used population-based Pennsylvania (PA) cancer registry data (PCR) from the PA Department of Health to examine the spatial-temporal pattern of survival in patients with a primary clinical diagnosis of PC in PA between 2004 and 2014 [5–8]. The PCR is annually collected, including demographic (e.g., age at diagnosis, race, insurance) and clinical information (e.g., serum prostate-specific antigen [PSA], Gleason score, tumor stage, first-course treatment) from hospitals, clinics, and other medical facilities, as well as geo-spatial information [4–8]. Note that in PA, there are around 78,000 newly diagnosed cancer cases each year, with a mortality rate of 169.1 per 100,000 men in 2004–2014 (age-adjusted to the 2000 US standard population) according to a PA Department of Health report on the burden of cancer in PA in 2019; however, there have been limited studies on PA survival on prostate cancer taking spatial heterogeneity and temporal trend into account.

**Fig. 1:**
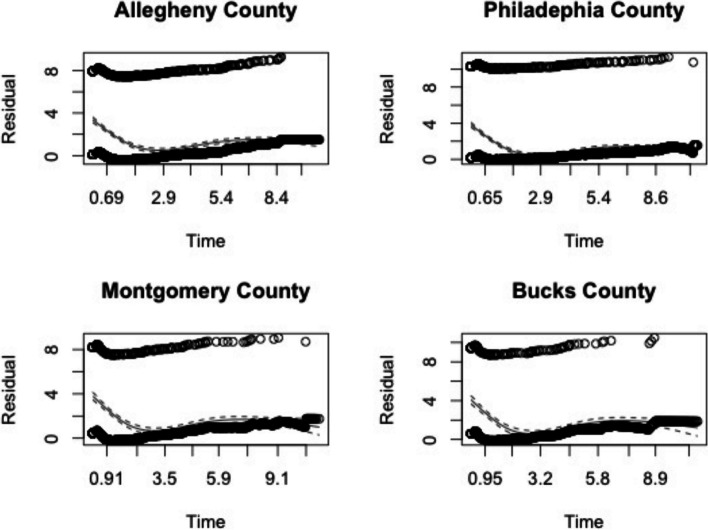
The plots of the Schoenfeld residuals for the PH assumption test on race

To analyze such registry data more efficiently for valid inference, advanced statistical methods for cancer survival analysis are needed. There are two widely used methods for time-to-event analysis: the Cox proportional hazards (PH) model and the accelerated failure time (AFT) model, both with extensive extensions [9–12]. The PH assumption is highly likely to be violated in cancer registry data owing to the multi-modality (i.e., demographic and clinical information) and hierarchical structure (i.e., individual-level, county-level) of risk factors [3, 8]. We performed a preliminary check of PCR data and realized that the plots of the Schoenfeld residuals indicated that the PH assumption was violated for race in several counties, as shown in Fig. 1. Owing to these specific data features and issues, Cox PH regression may lead to biased estimates and invalid inference. Additionally, failing to account for spatial-temporal heterogeneity could lead to biased inference; although substantial work has been done to address this issue, it has mostly focused on aggregate data analysis [13, 14]; individual-level spatial-temporal analysis is yet to be explored. Therefore, to overcome these barriers, advanced statistical models are needed for rigorous exploration. Here, we propose advanced spatial-temporal models under the AFT framework to fill this gap. Among the existing literature on cancer registry survival analysis, there has been some work performed in the realm of spatial or spatio-temporal survival analysis. Carlin and Banerjee (2003) [15] considered hierarchical spatial process models for multivariate survival datasets that are spatio-temporally arranged and used Cox PH modeling approaches with spatial and temporal effects; Banerjee and Carlin (2003) [16] later proposed a semi-parametric (i.e., Cox PH) hierarchical Bayesian frailty model to capture spatio-temporal heterogeneity for discrete survival times; Zhang and Lawson (2011) [3] proposed a spatial AFT model (with only spatial random effects considered); Zhou et al. (2017) [17] proposed a generalized AFT model with spatial frailty and considered informative censoring data; Onicescu et al. (2017) [18] developed a geographically augmented survival model with a complex spatio-temporal structure; however, their spatial and temporal components were not easily separated for interpretation; and Carroll et al. (2017) [19] proposed Bayesian AFT models with only spatial frailty terms to investigate spatial differences in breast cancer mortality following cancer diagnosis using the 2000–2013 Louisiana SEER data. Later, Wang et al. (2020) [8] used this approach to investigate the effects of risk factors on overall survival in newly diagnosed PC patients, and Carroll et al. (2019) [20] extended Bayesian AFT models to explore spatial and temporal options for structuring frailties that follow a random walk process, but they considered the AFT models with a standard logistic distribution for the error term and did not clearly mention how to better handle the variable of diagnosis year in regression analysis. Some other work includes Wang et al. (2012) [21], Hurtado et al. (2016) [22], Sharmin and Khan (2017) [23], Carroll and Zhao (2019) [24], among others. Comparing to the previous work, our proposed model has the following advantages. First, Bayesian AFT models are employed instead of Cox PH models and relax the PH assumption, and also our program possess different choices of distributions (e.g., Weibull, log-logistic); Second, the fixed effects including linear predictors of interest and the random effects with flexible frailty structures in space and time are incorporated. In particular, we explore different ways to handle the time variable (e.g., the year of diagnosis) which are under-studied in the literature, and evaluate their empirical performance of effectiveness on survival inference under a variety of settings; Third, we implement our estimation procedures and model diagnosis in R with callable C functions for computing efficiency, which are available in the GitHub for public use by cancer researchers and other research purposes (refer to https://github.com/zli141/sptime).

The rest of the paper is organized as follows. In the “Methods” section, we provide notation and describe our proposed spatial-temporal AFT models, as well as the Bayesian algorithm for parameter estimation and inference. The “Simulation study” section details the extensive simulations conducted to evaluate our proposal. The results from the motivating example of the PCR of PC are presented in the “Data application” section. Finally, the “Discussion” section offers discussion with concluding remarks and potential future work.

## Methods

### Accelerated failure time model

For the $k^{th}$ ($k=1,\ldots , K_{ij}$) subject from the $i^{th}$ ($i=1,\ldots ,I$) county in the $j^{th}$ ($j=1,\ldots ,J$) temporal cohort, let $T_{ijk}$ denote the time to death after diagnosis of PC, and let $C_{ijk}$ represent the corresponding censoring time. Thus, $Y_{ijk}=\min (T_{ijk},C_{ijk})$ is the observed follow-up time, with $\delta _{ijk}=I(T_{ijk} \le C_{ijk})$ as the death indicator. In addition, $\varvec{x}_{ijk}$ is a $p \times 1$ vector of covariates for survival regression, which could include time-dependent or time-varying factors of interest.

The AFT model can be expressed in a linear form with the log link function of $T_{ijk}$,

1 $$\begin{aligned} \log (T_{ijk})=\mu +\varvec{x}^T_{ijk}\varvec{\beta }+\sigma \epsilon _{ijk}, \end{aligned}$$

where $\mu$ is the population-level mean and $\sigma$ is a shape parameter that controls the shape of the survival curve; $\varvec{\beta }$ is a $p \times 1$ vector of regression coefficients associated with the covariates $\varvec{x}_{ijk}$; and $\epsilon _{ijk}$ is the residual, which follows a distribution function $F_\epsilon (\cdot )$. For instance, we can consider an $\epsilon _{ijk}$ that follows a standard extreme value distribution; thus, $f_{\epsilon }(\varepsilon )=\exp (\varepsilon -e^\varepsilon )$ and $F_\epsilon (\varepsilon )=1-\exp (-e^{\varepsilon })$, where $\epsilon$ follows a standard extreme value distribution and so $T_{ijk}$ follows a Weibull distribution. Other choices for $\epsilon _{ijk}$ include a standard normal distribution and a standard logistic distribution.

### The conditional autoregressive (CAR) prior

In order to account for the county level spatial heterogeneity, we include a spatial random effect in the AFT model, which is given by

2 $$\begin{aligned} \log (T_{ijk})=\mu +\varvec{x}^T_{ijk}\varvec{\beta }+\omega _i +\sigma \epsilon _{ijk}. \end{aligned}$$

Borrowing an idea from linear mixed-effect models, we could assume that the random effect $\omega _i$ follows a normal distribution $N(0,\tau ^{-2}).$ However, unlike traditional mixed-effect models, the $\omega _i$ $(i=1,\ldots , I)$ are not independently distributed; this is because the adjacent counties tend to be correlated owing to the potential of sharing similar environmental or social factors [25]. Besag (1974) [26] proposed a conditional autoregressive (CAR) distribution for $\omega _i$ and assumed

3 $$\begin{aligned} \omega _i|\varvec{\omega }_{-i}\sim N\left( \frac{\sum _{i' \ne i} m_{ii'} \omega _{i'}}{\sum _{i'\ne i} \omega _{ii'}}, \frac{1}{\sum _{i'\ne i} m_{ii'}}\tau ^{-2}\right) , \end{aligned}$$

where $\varvec{\omega }_{-i}$=$\{\omega _1,\ldots ,\omega _{i-1},\omega _{i+1},\ldots ,\omega _{I}\}$. Note that $m_{ii'}$ is defined as 1 if county *i* and county $i'$ are adjacent; otherwise $m_{ii'}=0.$ This can be interpreted as the arithmetic mean of $\omega _i$ being the arithmetic mean of the $\omega _{i}$ values of those counties adjacent to it. The joint density function of $\varvec{\omega }=(\omega _1,\dots ,\omega _I)^{T}$ can be expressed by

4 $$\begin{aligned} \textrm{Pr}(\varvec{\omega })\propto \tau ^{-n}\exp \left(-\frac{1}{2\tau ^2}\varvec{\omega }^T(\varvec{D}_\omega -\varvec{C})\varvec{\omega }\right), \end{aligned}$$

where $\varvec{D}_\omega =\text {diag}\left\{ \sum _{i' \ne 1}m_{1i'},\dots , \sum _{i'\ne I}m_{Ii'}\right\}$ is a diagonal matrix, with the $i^{th}$ diagonal element as the total number of the counties adjacent to county *i*. $\varvec{C}=(m_{ii'})_{I\times I}$ is the adjacent matrix of all the counties in the study.

Given the observed data $D =\{(Y_{ijk},\delta _{ijk}), i=1,\ldots ,I, j=1,\ldots ,J, k=1,\ldots , K_{ij}\}$ and spatial random effects $\varvec{\omega }$, the conditional likelihood function can be derived as

5 $$\begin{aligned} L(D|\varvec{w})=\prod _{i=1}^{I}\prod _{j=1}^{J}\prod _{k=1}^{K_{ij}}\left[ f(t_{ijk}|\omega _i)\right] ^ {\delta _{ijk}} \left[ S(t_{ijk}|\omega _i)\right] ^{1-\delta _{ijk}}, \end{aligned}$$

where $f(t_{ijk}|\omega _i)$ and $S(t_{ijk}|\omega _i)$ are the conditional density function and survival function, respectively, for the $k^{th}$ subject from the $i^{th}$ county in the $j^{th}$ temporal cohort. Denoting $\mu (\varvec{x}_{ijk})=\mu +\varvec{x}_{ijk}^T \varvec{\beta }+\omega _i$, we have

6 $$\begin{aligned} S(t_{ijk}|\omega _i)=S_{\epsilon }\left( \frac{\log (t_{ijk})-\mu (\varvec{x}_{ijk})}{\sigma }\right) = \exp \left[ -\exp \left( \frac{\log (t_{ijk})-\mu (\varvec{x}_{ijk})}{\sigma }\right) \right] , \end{aligned}$$

and

7 $$\begin{aligned} f(t_{ijk}|\omega _i)=1/(\sigma t_{ijk})f_{\epsilon }\left( \frac{\log (t_{ijk})-\mu (\varvec{x}_{ijk})}{\sigma }\right) . \end{aligned}$$

### The multivariate conditional autoregressive (MCAR) prior

The CAR prior only accounts for the correlations between different counties; however, the PCR for PC also includes patients enrolled in different years (i.e., temporal cohorts). It is natural to anticipate that there might be some degree of correlation between either temporal cohorts or counties. Banerjee and Carlin (2003) [16] proposed a Cox PH model with the MCAR prior to address the spatial-temporal dependency. Owing to the violation of the PH assumption (Fig. 1), we extended the application of the MCAR prior to the AFT model for further survival analysis [27, 28].

Here, we induce another random effect vector regarding temporal cohorts of the $i^{th}$ county, $\varvec{\gamma }_i=(\gamma _{i1},\dots , \gamma _{iJ})^{T}$, into the AFT model. The spatial-temporal model can be expressed as

8 $$\begin{aligned} \log (T_{ijk})=\mu +\varvec{x}_{ijk}^{T}\varvec{\beta }+\varvec{z}_{ik}^{T}\varvec{\xi }+\varvec{\eta }_{i}^{T}\varvec{\gamma }_i+\omega _i+\sigma \epsilon _{ijk}, \end{aligned}$$

where $\varvec{z_{ik}}=(z_{i1k},\dots ,z_{iJk})^{T}$ is a $J \times 1$ with $z_{ijk}$ as a dichotomous temporal cohort indicator (1=yes, 0=no) for the $k^{th}$ patient from the $i^{th}$ county; $\varvec{\xi }= (\xi _1,...,\xi _J)^T$ is the fixed temporal effect; $\varvec{\eta _{i}}=(\eta _{i1},\dots ,\eta _{iJ})^{T}$ with $\eta _{ij}$ as a binary indicator covariate associated with the $j^{th}$ year-specific random effect $\gamma _{ij}$.

Let $\varvec{\Delta }_i=(\omega _i, \varvec{\gamma }_i^{T})^{T}$, and we assume

$$\begin{aligned} \varvec{\Delta }_i\sim N(\varvec{0}, \varvec{\Lambda }^{-1}) \end{aligned}$$

$$\begin{aligned} \varvec{\Delta }_i|\varvec{\Delta }_{-i} \sim MVN\Big (\frac{\sum _{i \ne i'} m_{ii'} \varvec{\Delta }_{i'}}{\sum _{i\ne i'} m_{ii'}}, \frac{1}{\sum _{i\ne i'} m_{ii'}}\varvec{\Lambda }^{-1}\Big ), \end{aligned}$$

where $\varvec{\Lambda }$ represents the hyper-parameters in the MCAR prior. Thus, the joint density function of $\varvec{\Delta }=\text {vec}\left\{ \varvec{\Delta }_{1},\dots , \varvec{\Delta }_I\right\}$ is

$$\begin{aligned} \textrm{Pr}(\varvec{\Delta }) \propto |\varvec{\Lambda }|^{\frac{1}{2}} \exp \Big (-\frac{1}{2}\varvec{\Delta }^T \left[ (\varvec{D}_\omega -\varvec{C})\otimes \varvec{\Lambda }\right] \varvec{\Delta }\Big ). \end{aligned}$$

Note that the MCAR prior may be improper, because the variance-covariance matrix of the normal distribution could be singular. Similarly, the conditional likelihood of the observed data $D=\{(Y_{ijk},\delta _{ijk}), i=1,\ldots ,I, j=1,\ldots ,J, k=1,\ldots ,K_{ij}\}$ is

9 $$\begin{aligned} L(D|\varvec{\Delta })=\prod _{i=1}^{I}\prod _{j=1}^{J}\prod _{k=1}^{K_{ij}}\left[ f(t_{ijk}|\varvec{\Delta }_i)\right] ^ {\delta _{ijk}} \left[ S(t_{ijk}|\varvec{\Delta }_i)\right] ^{1-\delta _{ijk}}, \end{aligned}$$

where $f(t_{ijk}|\varvec{\Delta }_i)$ and $S(t_{ijk}|\varvec{\Delta }_i)$ are the conditional density function and survival function, respectively, for the $k^{th}$ subject from the $i^{th}$ county in the $j^{th}$ temporal cohort. Denote $\mu '(\varvec{x}_{ijk})=\mu +\varvec{x}_{ijk}^{T}\varvec{\beta }+\varvec{z}_{ik}^{T}\varvec{\xi }+\varvec{\eta }_{i}^{T}\varvec{\gamma }_i+\omega _i$, we have

10 $$\begin{aligned} S(t_{ijk}|\varvec{\Delta }_i)=S_{\epsilon }\left( \frac{\log (t_{ijk})-\mu '(\varvec{x}_{ijk})}{\sigma }\right) = \exp \left[ -\exp \left( \frac{\log (t_{ijk})-\mu ' (\varvec{x}_{ijk})}{\sigma }\right) \right] , \end{aligned}$$

and

11 $$\begin{aligned} f(t_{ijk}|\varvec{\Delta }_i)=1/(\sigma t_{ijk})f_{\epsilon }\left( \frac{\log (t_{ijk})-\mu '(\varvec{x}_{ijk})}{\sigma }\right) . \end{aligned}$$

### Bayesian inference with the MCAR prior

When $\varvec{D}_\omega -\varvec{C}$ is a non-singular matrix, the density function of $\varvec{\Delta }$ is proper, and we can find a unique solution based on the likelihood approach. However, when $\varvec{D}_\omega -\varvec{C}$ is a singular matrix, the density function is not proper; thus, Bayesian methods are preferred for parameter estimation and inference. Here, we consider the non-informative priors for $\varvec{\beta }$, $\varvec{\xi }$, $\mu$, and $\sigma ^2$, where

$$\begin{aligned} \textrm{Pr}(\varvec{\beta })\propto 1, ~~\Pr (\varvec{\xi }) \propto 1,~~ \mu \propto 1, ~~\sigma ^2 \sim \text {inverse-Gamma} (0.001,0.001). \end{aligned}$$

Also, with regard to random effects $\varvec{\Delta }_i$, we select a conjugated prior for $\varvec{\Lambda }$ with $\varvec{\Lambda }\sim \text {Wishart} \left( p,\varvec{R}\right) .$ In order to let the prior be vague, *p* could be the dimension of $\varvec{\Lambda },$ and $\varvec{R}$ could be arbitrarily set as a diagonal matrix $Diag\{100,...,100\}_{I\times I}.$ Let $\varvec{X}=\{\varvec{x}_{ijk}\}$, $\varvec{Z}=\{z_{ijk}\}$; $i=1,\ldots ,I$; $j=1,\ldots ,J$; $k=1,\ldots , K_{ij}$. Then, we can derive the posterior distribution of $\varvec{\Lambda }$ as follows:

$$\begin{aligned} \varvec{\Lambda }|D,\varvec{\beta },\varvec{\Delta }, \sigma \sim \text {Wishart}(p+I, (\varvec{R}^{-1}+\varvec{V})^{-1}), \end{aligned}$$

where the element in the $i^{th}$ row and $j^{th}$ column of $\varvec{V}$ is $V_{ij}=\varvec{\Delta }_i^{*T}(\varvec{D}_w-\varvec{C})\varvec{\Delta }^{*}_j$, with $\varvec{\Delta }_i^{*}=(\Delta _{1i},\dots ,\Delta _{Ii})^T$ and $\varvec{\Delta }_j^{*}=(\Delta _{1j},\dots ,\Delta _{Ij})^T$. The Gibbs sampler algorithm is used to generate the posterior samples of $\varvec{\beta }$, $\varvec{\xi }$, $\mu$, $\sigma$, and $\varvec{\Lambda }$ [29]. In particular, for the $t^{th}$ iteration, $t=1,\ldots , M$ (where *M* is the total number of samples we will draw from the posterior distribution), we have the following:

Step 1: Sample $\varvec{\beta }^{(t)}$ from $\textrm{Pr}(\varvec{\beta }^{(t)}|D,\varvec{X,Z},\varvec{\xi }^{(t-1)},\varvec{\Delta }^{(t-1)}, \sigma ^{(t-1)},\varvec{\Lambda }^{(t-1)})$;
Step 2: Sample $\varvec{\xi }^{(t)}$ from $\textrm{Pr}(\varvec{\xi }^{(t)}|D,\varvec{X,Z},\varvec{\beta }^{(t)},\varvec{\Delta }^{(t-1)}, \sigma ^{(t-1)},\varvec{\Lambda }^{(t-1)})$;
Step 3: Sample $\varvec{\omega }_i^{(t)}$ from $\textrm{Pr}(\varvec{\Delta }_i^{(t)}|D,\varvec{X,Z},\varvec{\beta }^{(t)},\varvec{\omega }_{(-i)}^{(t-1)}, \sigma ^{(t-1)},\varvec{\Lambda }^{t-1})$, for $i=1,\dots ,I$;
Step 4: Sample $\sigma ^{(t)}$ from $\textrm{Pr}(\sigma ^{(t)}|D,\varvec{X,Z},\varvec{\beta }^{(t)},\varvec{\xi }^{(t)},\varvec{\Delta }^{(t)}, \varvec{\Lambda }^{(t-1)})$;
Step 5: Sample $\varvec{\Lambda }^{(t)}$ from $\textrm{Pr}(\varvec{\Lambda }^{(t)}|\varvec{D,X,Z},\varvec{\beta }^{(t)},\varvec{\xi }^{(t)},\varvec{\Delta }^{(t)}, \sigma ^{(t)})$.

Of note, there is no closed form for the full conditional posterior distribution except in step 5, which is a Wishart distribution, $\text {Wishart}(p+I, (\varvec{R}^{-1}+\varvec{V}^{-1})^{-1})$. The Metropolis–Hastings algorithm was used to sample the parameters from their full conditional distribution [30]. Taking $\textrm{Pr}(\varvec{\beta }^{(t)}|D,\varvec{X,Z},\varvec{\xi }^{(t-1)},\varvec{\Delta }^{(t-1)}, \sigma ^{(t-1)},\varvec{\Lambda }^{(t-1)})$ as an example, we have the following procedures:

1. Generate $$U\sim Unif(0,1)$$ and $$W\sim N(0,1)$$;
2. Generate $\varvec{\beta }^{New}$ from $\varvec{\beta }^{New}=\varvec{\beta }^{(t-1)}+sW$, where *s* is the step size of a random walk process;
3. Calculate
  $$\begin{aligned} LR=\frac{L(D|\varvec{\beta }^{New},\varvec{\Delta })\textrm{Pr}(\varvec{\beta }^{New})}{L(D|\varvec{\beta }^{(t-1)},\varvec{\Delta })\textrm{Pr}(\varvec{\beta }^{(t-1)})}; \end{aligned}$$
4. If $$LR>U,$$ $$\beta ^{(t)}=\beta ^{New}$$, otherwise $$\beta ^{(t)}=\beta ^{(t-1)}$$.

Here, *s* is chosen to ensure the rejection rate is not extremely high, given that an optimal acceptance rate would be between 10% and 60%. In our simulation, our rejection rate is around 44%; we repeatedly sample $\varvec{\beta },\varvec{\xi }, \mu$, $\sigma$ and $\varvec{\Lambda }$ from step 1 to step 5 until we have enough posterior samples. Suppose we have a total of *M* posterior samples; then, we can estimate the parameter by calculating their posterior means and also perform subsequent inference. To ensure the parameters in the model identifiable, $\Delta _{ij}$ will be centralized such that $\sum _{ij}\Delta _{ij}=0.$

### Model selection and goodness-of-fit check

With respect to model selection, we used the deviance information criteria (DIC), a Bayesian analog of the AIC, to choose the best candidate model that achieves the optimal balance between model fit and model complexity [31]. Let $\varvec{\theta }$ denote the whole parameter space of the model. Spiegelhalter et al. (2002) [31] proposed the following DIC under the Bayesian framework, which combines the likelihood and the posterior distributions:

12 $$\begin{aligned} DIC=2p_D+\zeta(\bar{\varvec{\theta }}), \end{aligned}$$

where $\bar{\varvec{\theta }}$ is the posterior mean of $\varvec{\theta }$. Noting that $\zeta(\bar{\varvec{\theta }})=-2\log L(\bar{\varvec{\theta }})+C$ is the deviance of the model under our posterior estimates, where *C* is a constant on the DIC; and $p_D=\bar{\zeta}-\zeta(\bar{\varvec{\theta }})$ is the difference of the mean of the deviance under the posterior distribution and the deviance under the posterior mean, reflecting the effective number of parameters to indicate the model complexity or degrees of freedom. Notably, DIC serves as a decent approximation of AIC when working with negligible prior information. Additionally, graphical and secondary assessments such as Cox-Snell or martingale residuals are valuable tools for gaining further insights into the goodness-of-fit.

Motivated by the analysis using the PCR of PC, we consider the following three candidate models:

- Model 1 (M1): $$\log (T_{ijk})=\mu +\varvec{x}_{ijk}^{T}\varvec{\beta }+\xi z_{ijk}+\omega _{i}+\sigma \epsilon _{ijk}$$;
- Model 2 (M2): $$\log (T_{ijk})=\mu +\varvec{x}_{ijk}^{T}\varvec{\beta }+\xi z_{ijk}+ \gamma _i z_{ijk}+\omega _{i}+\sigma \epsilon _{ijk}$$;
- Model 3 (M3): $$\log (T_{ijk})=\mu +\varvec{x}_{ijk}^{T}\varvec{\beta }+\varvec{z}_{ik}^{T}\varvec{\xi }+\varvec{\eta }_{i}^{T}\varvec{\gamma }_i+\omega _i+\sigma \epsilon _{ijk}.$$

In M1 and M2, $z_{ijk}$ is the year when the patient was enrolled in the study. These models treat the year as a continuous variable. In M3, $\varvec{z}_{ik}$ is the vector of the year indicators for the year when the patient was enrolled in the study, treating year as a categorical variable. M1 is a spatial model that does not account for the temporal cohort effect variation. In M2, a random slope is added to account for the temporal effect, where $\varvec{\Delta }_i=\{\omega _i,\gamma _i\}$. For M3, we have a spatial-temporal intercept for each cohort and each county $\varvec{\Delta }_i=\{\omega _{i}, \gamma _{ij}, i=1,\dots ,I; j=1,\dots ,J\}.$ We chose the optimal model for survival analysis of the PCR of PC depending on the DIC, where the smaller the DIC, the better the model. The procedures for parameter estimation and inference and for model diagnosis were programmed in R and invoke C functions; these are available upon request from the authors.

## Simulation study

### Simulation set-ups

In order to evaluate the performance of our proposed method and the selection accuracy of the DIC, we mimicked the PCR data structure and conducted extensive simulation studies under different scenarios. The data were generated using the following AFT models:

- Scenario 1 (S1): $$\log T_{ijk}=\mu + x_{1,ijk}+0.5x_{2,ijk}+\omega _i+0.5(j-1)+\epsilon _{ijk}$$;
- Scenario 2 (S2): $$\log T_{ijk}=\mu +x_{1,ijk}+0.5x_{2,ijk}+\omega _i+0.5(j-1)^{0.5}+\epsilon _{ijk}$$;
- Scenario 3 (S3): $$\log T_{ijk}=\mu + x_{1,ijk}+0.5x_{2,ijk}+\omega _{i}+\gamma _{ij}+\epsilon _{ijk}$$;
- Scenario 4 (S4): $$\log T_{ijk}=\mu +x_{1,ijk}+0.5x_{2,ijk}+\omega _{i}+\varvec{z}^T_{ik}\varvec{\xi }+\epsilon _{ijk}$$, where $$\varvec{\xi }=(0, 0.5, -0.5, 0.6, -0.8)$$.

Here, $x_{1,ijk}$ is a continuous variable generated from a standard normal distribution *N*(0, 1); $x_{2,ijk}$ is a binary variable following the Bernoulli distribution, *Bernoulli*(0.5); $x_{1,ijk}$ and $x_{2,ijk}$ vary between different subjects, cohorts, and counties; $\varvec{z}_{ik}=(z_{i1k}, \ldots , z_{iJk})$ with $z_{ijk}$ as the indicator for the $j^{th}$ temporal cohort. In S3, $\omega _{ij}=\omega _{i}+\gamma _{ij}$ is generated iteratively considering the dependency between adjacent cohorts with $\omega _{ij}=2\omega _{i,j-1}+\zeta$, where $\zeta$ is generated by a standard normal distribution *N*(0, 1). We assume that the error term $\epsilon _{ijk}$ follows a standard extreme value distribution.

To evaluate the influence of the censoring rate on the performance of our proposed candidate models, the censoring time was generated from a uniform distribution, *Unif*(0, 1) for a rough average censoring rate of 20% across Monte Carlo data, and *Unif*(0, 80) for a censoring rate of around 80%. Additionally, to mimic the PCR and generate similar data, we also considered the case with $x_{1,ijk}$ as a county-level risk factor to evaluate our methods; in other words, $x_{1,ijk}$ varies only across counties but not across subjects and temporal cohorts, thus $x_{1,ijk}=x_{1,i}$.

As there are 67 counties in PA, we considered the same number of counties in our set-up, thus $i=1,\ldots , 67$. In addition, in order to reduce the computing burden, we assumed there were five temporal cohorts for each county $(J=5)$. In each county and temporal cohort, the number of patients, $K_{ij}$, was determined by the percentage of cancer cases in the real PC data from the PCR. Thus, we ensured that there were at least five patients in every county each year. For results summary, we generated 1,000 Monte Carlo data for each scenario, and for each data simulation, we fitted the following Weibull AFT models (M1, M2, and M3) under the Bayesian framework:

- M1: $$\log (T_{ijk})=\mu +\beta _1 x_{1,ijk}+\beta _2 x_{2,ijk}+\omega _{i}+\sigma \epsilon _{ijk}$$;
- M2: $$\log (T_{ijk})=\mu +\beta _1 x_{1,ijk}+\beta _2 x_{2,ijk}+\xi z_{ijk}+\gamma _i z_{ijk}+\omega _{i}+\sigma \epsilon _{ijk}$$;
- M3: $$\log (T_{ijk})=\mu +\beta _1 x_{1,ijk}+\beta _2 x_{2,ijk}+\varvec{z}_{ik}^{T}\varvec{\xi }+\varvec{\eta }_{i}^{T}\varvec{\gamma }_i+\omega _{i}+\sigma \epsilon _{ijk}.$$

Note that $\varvec{\eta }_i$ and $\varvec{\gamma }_i$ are defined the same as in “Model selection and goodness-of-fit check” section, and the matrices $\varvec{D}_\omega$ and $\varvec{C}$ (the adjacency matrix) in the MCAR and CAR priors are generated for the PA counties. For parameter estimation, the posterior mean was calculated for each parameter using 1,000 MCMC samples, generating a total of 2,000 posterior samples with the first 1,000 samples discarded during the burn-in period. It should be noted that more MCMC samples might be necessary depending on model convergence diagnosis; however, in our numerical studies, satisfactory results were achieved under these settings (see results below). In summary, for the Monte Carlo replications, the bias (Bias) and standard deviation (SD) of parameter estimates, mean squared error (MSE), and the selection probabilities of different models based on the DIC are reported.

### Simulation results

The summary statistics for the estimates of primary parameters $\hat{\beta }_1$ and $\hat{\beta }_2$ under different scenarios are presented here. The results for all scenarios with a censoring rate of 20% are shown in Table 1. The bias values $\hat{\beta }_1$ and $\hat{\beta }_2$ under M2 were the smallest among all candidate models, whereas M1 had the worst performance with the largest bias, especially for S3, followed by S1. With regard to MSE, M2 still showed the best performance, having the smallest value across different scenarios. Notably, the performance of M3 was comparable with that of M2 in terms of having negligible bias and variability under the S4 scenario.

**Table 1:** Summary of the estimation results for the scenarios with the censoring rate is 20%. Par: parameters; Bias: the bias of Monte Carlo average of parameter estimates; SD: Monte Carlo standard deviation of parameter estimates; MSE: mean squared error

		S1			S2			S3			S4		
	Par	Bias	SD	MSE	Bias	SD	MSE	Bias	SD	MSE	Bias	SD	MSE
M1	$\beta _1=1$	-0.032	0.017	0.001	-0.016	0.015	0.000	-0.125	0.140	0.035	-0.013	0.015	0.000
	$\beta _2=0.5$	-0.015	0.031	0.001	-0.008	0.026	0.001	-0.062	0.082	0.010	-0.007	0.027	0.001
M2	$\beta _1=1$	0.000	0.011	0.000	0.000	0.010	0.000	-0.017	0.029	0.001	-0.004	0.012	0.000
	$\beta _2=0.5$	0.000	0.020	0.000	0.000	0.019	0.000	-0.009	0.029	0.001	-0.001	0.022	0.000
M3	$\beta _1=1$	-0.023	0.014	0.001	-0.009	0.012	0.000	-0.097	0.112	0.022	-0.003	0.012	0.000
	$\beta _2=0.5$	-0.011	0.024	0.001	-0.004	0.021	0.000	-0.047	0.062	0.006	-0.002	0.022	0.001

**Fig. 2:**
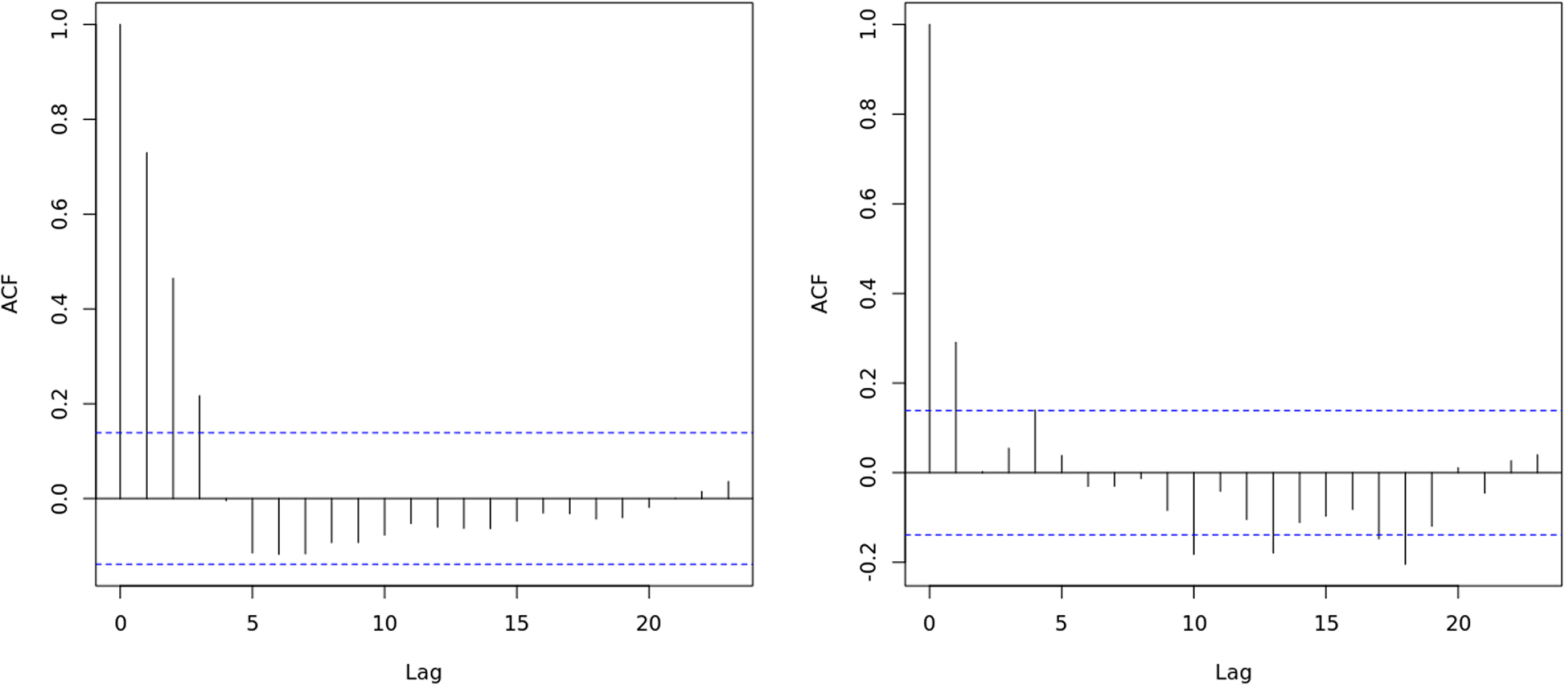
The ACF plot for model parameters of $\beta _1$ (left panel) and $\beta _2$ (right panel)

**Fig. 3:**
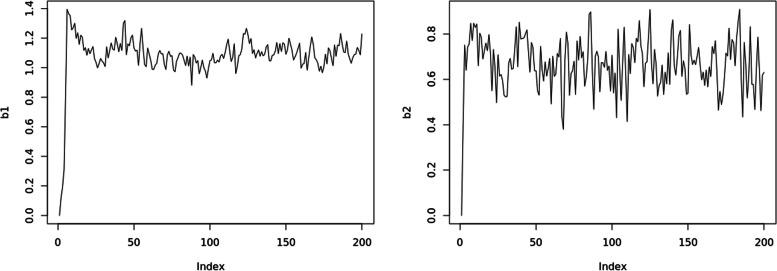
The trace plot for model parameters of $\beta _1$ (left panel) and $\beta _2$ (right panel)

Figures 2 and 3 show that in this algorithm, $\beta _1$ and $\beta _2$ quickly converge to the true value. The posterior samples are not quite correlated in the autocorrelation function (ACF) plot. Also, in the trace plot, the posterior samples vibrate around the true value of $\beta _1$ and $\beta _2$.

Furthermore, when the censoring rate increased to 80% (results shown in Table 2), the majority of bias and MSE values across different scenarios increased compared with those in Table 1; however, M2 still showed satisfactory results in terms of having the smallest bias and MSE among all candidate models, except in the case of S4, under which the bias values ($\hat{\beta }_1$ and $\hat{\beta }_2$) were relatively large, although the MSEs remained satisfactory (0.002 and 0.005 for M2; 0.003 and 0.005 for M3). In addition, we checked the model fitting for the scenarios with $x_1$ as the only risk factor varying across counties; the results are presented in Table 3. Compared with the results shown in Table 1, the bias of $\hat{\beta }_1$ in M2 tended to be much larger; under S4 in particular, a substantial bias was detected compared with the M3 case, although the MSEs seemed to be comparable. With regard to model selection under different scenarios, the selection probabilities based on the DIC criterion are provided in Table 4. Model M2 was predominantly selected regardless of the censoring rate; however, when underlying risk factors were hierarchical under scenario S4, which has spatial and temporal heterogeneity but the same linear temporal trend within-county, M3 had the highest selection rate. Noting that we also performed post-hoc analysis by adopting log-logistic AFT for model fitting and comparing the performance to the other existing approaches, and the results of these additional analyses are presented in the Supplementary Material.

**Table 2:** Summary of the estimation results for the scenarios with the censoring rate is 80%. Par: parameters; Bias: the bias of Monte Carlo average of parameter estimates; SD: Monte Carlo standard deviation of parameter estimates; MSE: mean squared error

		S1			S2			S3			S4		
	Par	Bias	SD	MSE	Bias	SD	MSE	Bias	SD	MSE	Bias	SD	MSE
M1	$\beta _1=1$	-0.026	0.069	0.005	-0.020	0.061	0.004	-0.019	0.092	0.009	-0.031	0.046	0.003
	$\beta _2=0.5$	-0.006	0.103	0.011	-0.002	0.093	0.009	-0.011	0.113	0.013	-0.014	0.070	0.005
M2	$\beta _1=1$	0.004	0.064	0.004	0.004	0.057	0.003	0.025	0.076	0.006	-0.007	0.041	0.002
	$\beta _2=0.5$	0.008	0.102	0.010	0.009	0.092	0.009	0.011	0.095	0.009	-0.002	0.069	0.005
M3	$\beta _1=1$	-0.054	0.068	0.008	-0.020	0.063	0.004	-0.051	0.125	0.018	-0.003	0.055	0.003
	$\beta _2=0.5$	-0.021	0.100	0.010	-0.002	0.093	0.009	-0.026	0.106	0.012	0.000	0.073	0.005

**Table 3:** Summary of the estimation results for the scenarios with the censoring rate is 20% and the county-level risk factor $x_{1,i}$. Par: parameters; Bias: the bias of Monte Carlo average of parameter estimates; SD: Monte Carlo standard deviation of parameter estimates; MSE: mean squared error

		S1			S2			S3			S4		
	Par	Bias	SD	MSE	Bias	SD	MSE	Bias	SD	MSE	Bias	SD	MSE
M1	$\beta _1=1$	-0.032	0.133	0.019	-0.017	0.134	0.018	-0.114	0.184	0.047	-0.015	0.134	0.018
	$\beta _2=0.5$	-0.016	0.028	0.001	-0.008	0.024	0.001	-0.057	0.085	0.011	-0.007	0.027	0.001
M2	$\beta _1=1$	-0.022	0.089	0.008	-0.025	0.090	0.009	-0.084	0.131	0.024	-0.020	0.080	0.007
	$\beta _2=0.5$	0.000	0.020	0.000	0.001	0.020	0.000	-0.006	0.030	0.001	-0.002	0.023	0.001
M3	$\beta _1=1$	-0.030	0.101	0.011	-0.016	0.101	0.010	-0.092	0.148	0.030	-0.009	0.101	0.010
	$\beta _2=0.5$	-0.012	0.022	0.001	-0.004	0.021	0.000	-0.045	0.063	0.006	-0.002	0.023	0.001

**Table 4:** Summary results of the selection probabilities for different models under different scenarios. CR: censoring rate. $x_{1,ijk}$ is a subject-level risk factor varied across counties and temporal years; $x_{1,i}$ is a county-level risk factor

	20% CR, $x_{1,ijk}$			80% CR, $x_{1,ijk}$			20% CR, $x_{1,i}$		
	M1	M2	M3	M1	M2	M3	M1	M2	M3
S1	0.00	1.00	0.00	0.00	1.00	0.00	0.00	1.00	0.00
S2	0.00	1.00	0.00	0.00	1.00	0.00	0.00	1.00	0.00
S3	0.00	0.97	0.03	0.00	0.99	0.01	0.00	0.95	0.05
S4	0.00	0.10	0.90	0.00	1.00	0.00	0.00	0.12	0.88

## Data application

We applied our proposed models to the PCR PC data for the years 2004–2014, with men aged $\ge 40$ years with a primary diagnosis of PC and Gleason score (GS) of $\ge 6$ included for analysis. PC cases with unknown GS were also considered if the tumor stage was $\ge$ T3. The survival outcome of interest was the time to all-cause mortality (months). Several important risk factors were considered: 1) serum PSA; 2) age at diagnosis with a threshold of 65 years old (1 if age $<65$, 0 otherwise); 3) insurance status (0=yes; 1=no); 4) Appalachian region, a geopolitical designation defined by the Appalachia Regional Commission that roughly follows the spine of the Appalachian mountains (https://www.arc.gov/appalachian_region); 5) disease aggressiveness: less aggressive PC (GS 6 or GS 7 $[3+4]$, tumor stage T1-T2, and no distant metastasis), or more aggressive PC (GS $\ge 7$ $[4+3]$, tumor stage $\ge$T3, or distant metastasis); 6) treatment at diagnosis: primary site surgery only, radiation only, primary site surgery and radiation, or other/unknown; 7) race: white, black, or other/unknown; and 8) year of diagnosis. Regarding the age variable, we dichotomized age using a threshold of 65 years due to clinical interest and significance [32–35], and also the violation of the PH assumption (Table S.3 in the Supplementary Material). The PA Department of Health and the Institutional Review Board of the PA State University College of Medicine approved the protected data and the study. Based on the empirical performance of the candidate models in the simulation studies, we only fitted two models, M2 and M3, for data analysis and comparison of results.

**Fig. 4:**
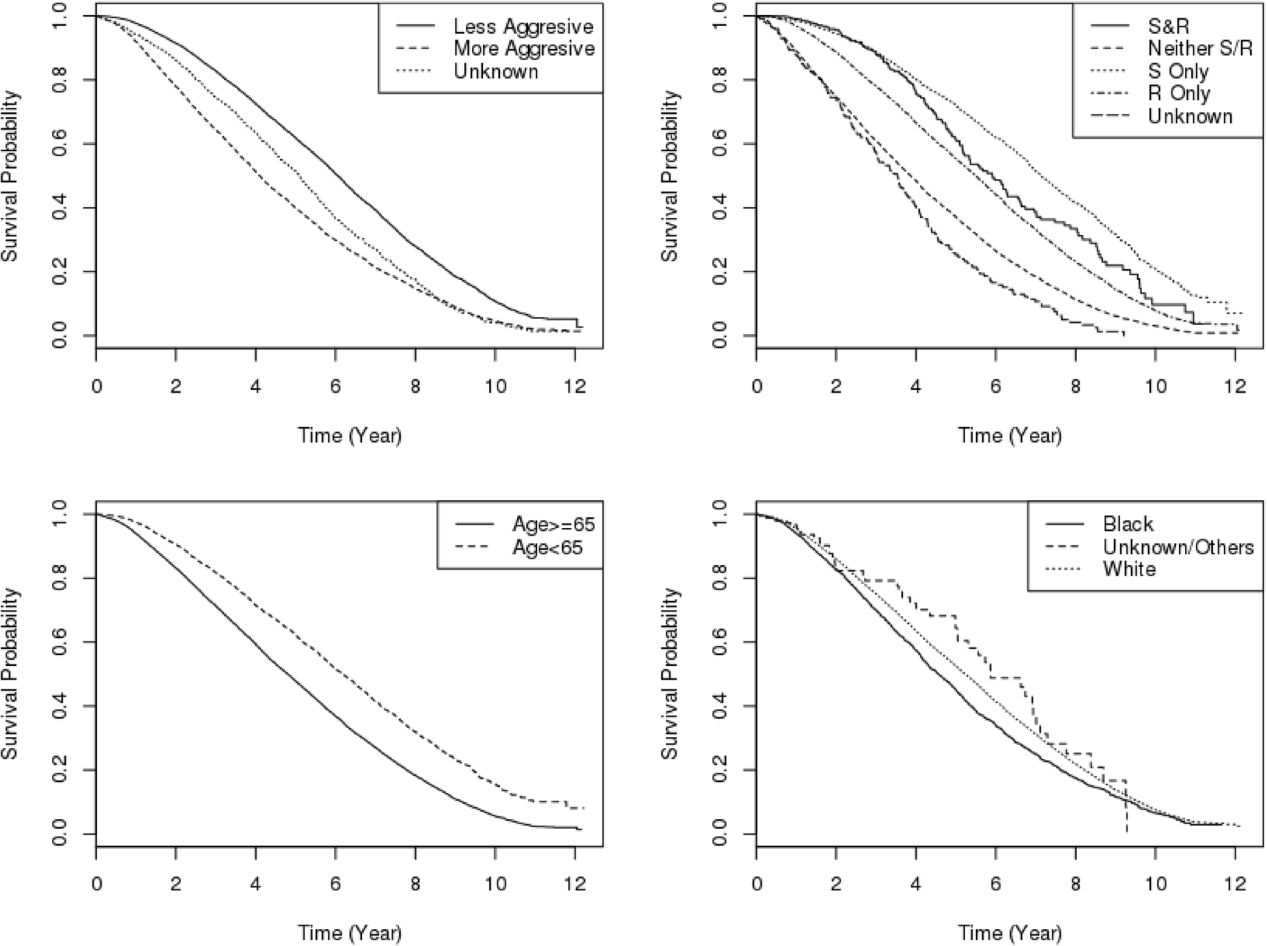
The Kaplan-Meier Curves stratified by stage of aggressiveness, first-course treatment (S: surgery; R: radiation), age and race. The p-values are obtained based on log-rank tests, where the category of unknown or unknown/others are not considered. Note: all *p*-values < 0.001 based on log-rank tests

Of the 143,499 PC cases reported with a primary diagnosis in the 2004–2014 PCR, 94,274 eligible men, aged 40 to 105 years, were identified for the final analysis. These data were analyzed in our previous work, where more details of the summary statistics for demographic and clinical characteristics can be found [6, 8]; however, these previous studies did not consider temporal heterogeneity together with spatial information in the modeling, nor were any model diagnoses or comparisons conducted. Here, we performed a secondary analysis of the PCR to further explore the distribution of newly diagnosed PC cases and their associated risk factors in PA by using advanced statistical methods via AFT regression models. As shown in Fig. 4, there were significant differences in the survival curves stratified by several risk factors ($p<0.001$). For example, patients who were black, had more aggressive disease, were aged 65$+$, or did not receive either surgery or radiation had higher risk of mortality. Based on an empirical check of the different candidate models in the simulations, we considered spatial-temporal AFT models M2 and M3 with adjustment of both spatial and temporal heterogeneity for this PCR data application; two different distribution assumptions, the Weibull and log-logistic distributions, were also considered. For each model, 2000 samples were drawn in the burn-in period and another 20000 samples were drawn from the posterior distribution.

The results for fixed-effect parameters are summarized in Table 5. Note that the estimates were directly associated with the natural logarithm of time, with a negative value indicating a decrease in survival time and a positive value indicating an increase in survival time. There were some differences in the magnitude of parameter estimates and the associated significance among different candidate models. Based on the DIC criterion, M3 with the log-logistic distribution assumption was the best candidate model. In addition, per reviewers’ suggestion, we conducted sensitivity analysis with varied values of hyperparameters for M3. Specifically, several combinations of priors for $\sigma ^2$ and ${\textbf {R}}$ were considered (with more details in the Supplementary Material). Based on the DIC, we added the best candidate model M3 with the priors $\sigma ^2\sim IG(0.001, 0.001)$ and ${\textbf {R}}=Diag{10,\dots ,10}$, denoted by M3 into Table 5. Overall, a significantly lower PC-specific survival time was observed for patients who were black, aged 65 and above, not insured, and had higher serum PSA or more aggressive PC at the time of diagnosis. A longer PC-specific survival time was observed for patients with any definitive PC treatment compared with those without either primary site surgery or radiation treatment. For instance, according to M3^∗^ (the best candidate), the average survival time of PC cases who received both surgery and radiation was 3.773=exp(1.328) (95% CI 3.695-4.536) times higher than that of those who did not receive either. When comparing models M3^∗^ to M3 under the log-logistic distribution assumption, one major difference is observed in the effect of the county-level risk factor, Appalachia. Notably, along with relatively informative priors, Appalachian regions exhibit longer survival times compared to non-Appalachian regions, which aligns with some evidence from our prior work (i.e., men residing in rural Appalachia demonstrated the lowest rates of aggressive prostate cancer and mortality) [8]. Nevertheless, this finding remains contentious and necessitates further exploration. Additionally, we conducted further goodness-of-fit checks using Cox-Snell residuals, with more detailed information available in the Supplementary Material (Fig. S.2). This analysis demonstrates superior performance after adjustment for spatial-temporal dependency, although we acknowledge some deviations that merit further investigation.

**Table 5:** Results summary for the data application of the PCR under M2 and M3 with Weibull and log-logistic AFT models. Note that the priors with $\sigma ^2\sim IG(0.001, 0.001)$ and ${\textbf {R}}=Diag\{100,\dots ,100\}$ are used except that for M3^∗^ with $\sigma ^2\sim IG(0.001, 0.001)$ and ${\textbf {R}}=Diag\{10,\dots ,10\}$. EST: parameter estimate; CL: credible limit; IG: inverse gamma

	Weibull AFT		Log-logistic AFT		
	M2	M3	M2	M3	M3^∗^
Covariate	EST (95%CL)	EST (95%CL)	EST (95%CL)	EST (95%CL)	EST (95%CL)
**Serum PSA**	-0.167(-0.180, -0.152)	-0.155(-0.177, -0.121)	-0.202(-0.218, -0.179)	-0.203(-0.236, -0.167)	-0.229 (-0.232, -0.190)
**Age at diagnosis**					
>=65	REF	REF	REF	REF	REF
<65	0.386(0.352, 0.414)	0.395(0.359, 0.428)	0.460(0.403, 0.494)	0.450(0.401, 0.489)	0.474 (0.472, 0.543)
**Insurance**					
Yes	REF	REF	REF	REF	REF
No	0.057(0.009, 0.097)	-0.092(-0.155, -0.013)	0.036(-0.026, 0.088)	-0.167(-0.235, -0.069)	-0.151 (-0.142, -0.030)
**Appalachian**					
No	REF	REF	REF	REF	REF
Yes	0.106(-0.068, 0.418)	-0.031(-0.153, 0.073)	0.061(-0.092, 0.547)	-0.107(-0.165, -0.081)	3.671 (4.003, 6.009)
**Treatment at diagnosis**					
Neither Surgery or Radiation	REF	REF	REF	REF	REF
Surgery only	0.974(0.909, 1.039)	0.978(0.917, 1.043)	1.097(1.049, 1.175)	1.106(1.054, 1.168)	1.152 (1.129, 1.237)
Radiation only	0.446(0.408, 0.483)	0.453(0.412, 0.493)	0.588(0.558, 0.616)	0.571(0.542, 0.614)	0.607 (0.610, 0.687)
Surgery and Radiation	1.071(0.963, 1.205)	1.015(0.911, 1.158)	1.279(1.077, 1.471)	1.240(1.081, 1.399)	1.328 (1.307, 1.512)
Others/Unknown	-0.246(-0.352, -0.125)	-0.102(-0.188, 0.054)	-0.312(-0.457, -0.101)	-0.224(-0.448, -0.057)	-0.294 (-0.281, -0.129)
**Race**					
White	REF	REF	REF	REF	REF
Black	-0.109(-0.150, -0.068)	-0.217(-0.285, -0.123)	-0.116(-0.170, -0.044)	-0.184(-0.234, -0.114)	-0.150 (-0.140, -0.061)
Others/Unknown	0.601(0.497, 0.696)	0.537(0.322, 0.724)	0.644(0.459, 0.809)	0.665(0.563, 0.784)	0.712 (0.710, 0.868)
**Stage of Aggressiveness**					
Less Aggressive	REF	REF	REF	REF	REF
More Aggressive	-0.435(-0.469, -0.408)	-0.453(-0.516, -0.409)	-0.528(-0.554, -0.499)	-0.525(-0.553, -0.486)	-0.593 (-0.594, -0.544)
Unknown	-0.117(-0.161, -0.071)	-0.129(-0.231, -0.057)	-0.117(-0.202, -0.068)	-0.072(-0.136, -0.017)	-0.155 (-0.138, -0.071)
**Year(Cont)**	0.044(0.039, 0.052)	-	0.075(0.068, 0.086)	-	-
Year=2004	-	REF	-	REF	REF
Year=2005	-	-0.661(-1.413, -0.056)	-	-0.036(-0.107, 0.078)	-1.034 (-1.076, -0.099)
Year=2006	-	0.106(-0.084, 0.224)	-	-0.114(-0.157, 0.022)	-0.374 (-0.419, -0.090)
Year=2007	-	-0.192(-0.495, 0.131)	-	-0.112(-0.165, 0.037)	-1.091 (-1.155, -0.361)
Year=2008	-	-0.529(-0.900, -0.221)	-	-0.103(-0.165, 0.050)	-1.706 (-1.782, -0.620)
Year=2009	-	0.095(-0.570, 0.781)	-	-0.053(-0.159, 0.110)	0.453 (0.474, 0.776)
Year=2010	-	0.416(-0.026, 0.741)	-	0.052(-0.030, 0.191)	0.977 (0.993, 1.244)
Year=2011	-	1.526(0.761, 1.891)	-	0.277(0.193, 0.407)	2.028 (1.653, 3.679)
Year=2012	-	1.495(0.754, 1.901)	-	0.267(0.193, 0.403)	1.997 (1.653, 3.633)
Year=2013	-	2.214(1.514, 2.996)	-	0.619(0.536, 0.765)	2.470 (2.242, 3.888)
Year=2014	-	5.375(2.499, 7.816)	-	1.271(1.018, 1.553)	5.167 (5.337, 7.368)
**Model Diagnosis**					
DIC	80202.51	89965.28	35360.85	34867.03	16397.44

## Discussion

In this work, we utilized PC data from the PCR to identify the best candidate model for inference. Owing to the unique features of individual-level cancer registry data, with spatial and temporal dependency and a hierarchical structure of risk factors, advanced statistical approaches via spatial-temporal hierarchical modeling were necessary. Although spatial and temporal models for survival analysis are described in the literature, they have limitations or were not directly applicable or accurate in this context: for instance, some approaches can only be used for aggregate data analysis such as county-level or any other administrative unit-level data; some only consider spatial correlations but ignore temporal information; and some use Cox PH regression regardless of the violation of the PH assumption. Based on our extensive simulation studies, mimicking the PCR and considering different candidate models to incorporate spatial and/or temporal heterogeneity, we identified an optimal model for cancer registry survival analysis with individual-level data under different scenarios. We found that in most cases, model M2 with spatial and temporal random effects and year of diagnosis as a continuous variable can achieve satisfactory performance, with the smallest bias and variability in parameter estimates. However, when there was substantial variation in space-time and also a hierarchical structure of risk factors across individuals and geographical clusters (i.e., county), model M3 was preferred. For goodness-of-fit check and model selection, the DIC is the most prevalent metric for evaluation. However, incorporating more graphical assessments like Cox-Snell residuals could provide additional insights. Moreover, based on sensitivity analysis, we anticipate that more informative priors might lead to further improvements. However, further exploration is required to ensure the justification of these priors.

These proposed models and the associated recommendation guidelines for model fitting under this context will benefit not only PC research but also studies of other cancers or diseases in other population-based registry data with similar data structures, including the SEER data, and the National Program of Cancer Registries. Furthermore, the Bayesian framework adopted here can be easily implemented in statistical software, and more interpretable results can be obtained from posterior summaries. Noting that if the model and data generating mechanism is unknown, the MCMC algorithm might take longer to converge depending on the model complexity and the data. Thus, it is important to perform convergence diagnosis and choose appropriate numbers of MCMC and burn-in samples. Nonetheless, there are several limitations to the proposed approach. Currently, we primarily focus on the CAR distribution, which incorporates spatial dependencies based on county contiguity. However, alternative weighting schemes such as distance-based or graphic-based weights could potentially offer greater insight. Additionally, enhancing the predictive accuracy of our approach could involve selecting more informative priors or other distribution assumptions tailored to specific data applications. Moreover, our current model only incorporates baseline risk factors except the diagnosis year. However, if additional (time-varying) risk factors are available for analysis, our modeling framework should be capable of accommodating them, albeit with additional effort required for algorithm extension and model diagnosis.

There are several topics for future work. Here, we mainly focused on parametric AFT models; however, the distribution assumption could be violated in practice, and thus more robust models such as the semi-parametric AFT model (i.e., rank-based estimation) would be of research interest. Besides, here we mainly analyzed overall survival; however, if there is clinical interest in cancer-specific survival, the competing risks due to other causes need to be taken into account to obtain unbiased estimates. Models extended from the Fine and Gray model or based on a joint approach with shared random effects modeling could be developed in future studies. In addition, with regard to parameter estimation and inference based on the Bayesian technique, advanced algorithms (i.e., blocked Gibbs sampling, slice sampling) could be adopted to further reduce the computing burden.

## Conclusion

This study presents several advanced spatial-temporal models for identifying risk factors for all-cause mortality in newly diagnosed PC patients from the PCR 2004-2014, where heterogeneity between subjects and the structure of dependency in geographic regions (county) and time (data collection year) are simultaneously considered. Simulation data indicated that under such context, the model with spatial and temporal random effects and the year of diagnosis as a continuous variable performs the best among candidate models, with model diagnosis and assessment based on the DIC. Additionally, the application of the model on our motivation data on PC was also evaluated, leading to more valid inference on risk factors’ effects and identifying substantial spatial-temporal variation.

### Supplementary Information

**Supplementary Material 1.**

## Data Availability

The Pennsylvania Cancer Registry (PCR) data: The PCR data is a population-based dataset including all newly diagnosed prostate cancer patients recorded by the Department of Health, Pennsylvania. The PCR data follows standardized data acquisition protocols to ensure that the individual reports include the same information in the same format, which can then be pooled in centralized databases. The PCR data can be made publicly available upon obtaining approval from the Pennsylvania Department of Health by signing a data use agreement for the data access application. The direct persistent weblink for the PCR data request is https://www.health.pa.gov/topics/Reporting-Registries/Cancer-Registry/Pages/Cancer%20Registry.aspx. Computing package: The computing package containing the code to perform spatial-temporal Bayesian accelerated failure time models described in the article. The package is written in R with callable C functions for computing efficiency, and it is available on GitHub for public use. Please refer to the website https://github.com/zli141/sptime.

## Abbreviations

PH: Proportional hazards
PC: Prostate cancer
AFT: Accelerated failure time
SEER: Surveillance, Epidemiology, and End Results Program
PA: Pennsylvania
PCR: Pennsylvania Cancer Registry
CAR: Conditional Autoregressive
MCAR: Multivariate Conditional Autoregressive
DIC: Deviance information criteria
SD: Standard deviation
MSE: Mean squared error
CR: Censoring rate
ACF: Autocorrelation function
GS: Gleason score
