# Supplementary material for: Spatial-temporal Bayesian accelerated failure time models for survival endpoints with applications to prostate cancer registry data
Source: BMC Med Res Methodol. 2024 Apr 8;24:86. doi: 10.1186/s12874-024-02201-w (PMC11003030; doi:10.1186/s12874-024-02201-w)

Supplementary Materials for "Spatial-Temporal Bayesian Accelerated Failure Time Models for Survival Endpoints with Applications to Prostate Cancer Registry Data"

Ming Wang [1,*], Zheng Li [2], Jun Lu [3], Lijun Zhang [1]
Yimei Li [4], Liangliang Zhang [1]

[1]Department of Population and Quantitative Health Sciences, Case Western Reserve University, Cleveland, OH, USA; [2]Novartis Pharmaceuticals, New Jersey, NJ, USA; [3]University of Illinois Chicago, Chicago, IL, USA; [4]Department of Biostatistics, Epidemiology, and Informatics, University of Pennsylvania, Philadelphia, PA, USA
*Contact Email: mxw827@case.edu

In this Supplementary Material, we present the additional simulation and application results. The rest of this material is organized as follows: Section 1 includes the additional simulation results; Section 2 displays additional results for the PCR data application.

1 Additional Simulation Results

We conducted further check on the performance of our proposal by considering the other distributions and also comparing to the other existing methods. First, we utilized similar simulation set-ups in the main body of the paper but considered the log-logistic distribution for survival data generation. Second, for comparison purpose, we considered a competing Cox regression model proposed by (1), where given the proportional hazard assumption holds, the hazard is modeled through the following three models:

- Model 1 (M1): $h(t_{ijk}) = h_{0i}(t_{ijk})h(\boldsymbol{x}_{ijk}^T\boldsymbol{\beta} + \xi z_{ijk} + \omega_i)$;
- Model 2 (M2): $h(t_{ijk}) = h_{0i}(t_{ijk})h(\boldsymbol{x}_{ijk}^T\boldsymbol{\beta} + \xi z_{ijk} + \gamma_i z_{ijk} + \omega_i)$;
- Model 3 (M3): $h(t_{ijk}) = h_{0i}(t_{ijk})h(\boldsymbol{x}_{ijk}^T\boldsymbol{\beta} + \boldsymbol{z}_{ik}^T\boldsymbol{\xi} + \boldsymbol{\eta}_i^T\boldsymbol{\gamma}_i + \omega_i)$.

We used the same Bayesian algorithms to fit the Cox PH model and the AFT model under the log-logistic distribution, with 200 samples for burn-in and 2000 samples generated from the posterior distribution. The overall results including the bias (Bias) of Monte Carlo average of parameter estimates, Monte Carlo standard deviation (SD) of parameter estimates, and mean squared error (MSE) are shown in Table S.1 (for the first scenario check) and Table

S.2 (for the second scenario check). We can see that our proposed models and program still worked satisfactory for the log-logistic AFT model fitting, and also achieved better performances than the existing models by (1) in terms of smaller bias and MSE when the data were generated from the log-logistic distribution.

2 Additional Results of Data Application

In this section, we provided the additional results for the PCR data analysis. In Table S.3, we get the results of PH tests for each covariate by using the Schoenfeld residuals against the transformed time, with p-value <0.05 showing the evidence of PH violation with additional evidences shown by county (Figure 1 in the main paper).

Per reviewers' suggestion, we also provided the Cox regression by (1) for comparison. We observe a consistent pattern and significant effects of the majority of risk factors on mortality. However, differences arise in the detection of significant effects for certain risk factors such as insurance, potentially due to the violation of the PH assumption (refer to Figure 1 in the main paper). Consequently, our primary focus remains on AFT models, and we do not provide the results for Cox regressions. Specifically, we assess survival curves (overall and by county) and examine the non-monotonic hazard function (estimated using the Epanechnikov boundary kernel function in the muhaz library) for the PCR, which are presented in Figures S.1 and S.2. Notably, we observe an initial increase followed by a decrease in the hazard rate, suggesting that the log-logistic distribution is a reasonable assumption. This finding aligns with the model selection based on the DIC criterion.

In addition, we considered several combinations of priors for σ^2 and $\mathbf{R}$. Based on the DIC, we can see the best candidate model with the priors $\sigma^2 \sim IG(0.001, 0.001)$ and $\mathbf{R} = Diag\{10, \ldots, 10\}$. It is noted that compared to the previous results of M3 (log-logistic), the significant effects of the risk factors remain to hold. Specifically, under this relatively more informative priors, the Appalachian regions tend to have longer survival than non-Appalachian regions, with some evidence detected in our previous work (i.e., men living in rural Appalachia had the lowest rates of aggressive prostate cancer and mortality, (2)). However, this finding is still controversial and warrants further exploration.

References

[1] Carlin, B.P., Banerjee, S.: Hierarchical multivariate car models for spatio-temporally correlated survival data. Bayesian statistics **7**, 45–63 (2003)

[2] Wang, M., Wasserman, E., Geyer, N., Carroll, R., Zhao, S., Hohl, R., Lengerich, E., McDonald, A.: Spatial patterns in prostate cancer-specific mortality in pennsylvania and its catchment area using pennsylvania cancer registry data, 2004-2014. BMC Cancer **20**(1), 394 (2020)

Table S.1: Summary of the estimation results for the scenarios with the censoring rate is 20% and model missepcification. Par: parameters; Bias: the bias of Monte Carlo average of parameter estimates; SD: Monte Carlo standard deviation of parameter estimates; MSE: mean squared error.

		S1			S2			S3			S4		
	Par	Bias	SD	MSE	Bias	SD	MSE	Bias	SD	MSE	Bias	SD	MSE
M1	$\beta_1 = 1$	-0.003	0.069	0.005	0.012	0.086	0.007	0.002	0.046	0.002	-0.013	0.015	0.000
	$\beta_2 = 0.5$	0.003	0.104	0.011	0.003	0.115	0.013	0.001	0.072	0.005	-0.007	0.027	0.001
M2	$\beta_1 = 1$	0.019	0.065	0.005	0.044	0.081	0.008	0.018	0.042	0.002	-0.004	0.012	0.000
	$\beta_2 = 0.5$	0.014	0.104	0.011	0.019	0.099	0.01	0.01	0.07	0.005	-0.001	0.022	0.000
M3	$\beta_1 = 1$	-0.035	0.068	0.006	0.005	0.135	0.018	0.031	0.053	0.004	-0.003	0.012	0.000
	$\beta_2 = 0.5$	-0.013	0.1	0.01	0	0.111	0.012	0.016	0.075	0.006	-0.002	0.022	0.001

Table S.2: Summary of the estimation results for the scenarios with the censoring rate is 20% and data generated by the log-logistic distribution. The models are fitted using the log-logistic distribution. Par: parameters; Bias: the bias of Monte Carlo average of parameter estimates; SD: Monte Carlo standard deviation of parameter estimates; MSE: mean squared error.

		S1			S2			S3			S4		
	Par	Bias	SD	MSE	Bias	SD	MSE	Bias	SD	MSE	Bias	SD	MSE
							Our Proposed Model						
M1	$\beta_1 = 1$	-0.003	0.020	0.000	-0.038	0.070	0.006	0.000	0.018	0.000	-0.013	0.015	0.000
	$\beta_2 = 0.5$	-0.001	0.036	0.001	-0.017	0.069	0.005	0.000	0.036	0.001	-0.007	0.027	0.001
M2	$\beta_1 = 1$	0.001	0.017	0.000	-0.012	0.033	0.001	0.001	0.017	0.000	-0.004	0.012	0.000
	$\beta_2 = 0.5$	0.000	0.033	0.001	-0.006	0.043	0.002	0.001	0.034	0.001	-0.001	0.022	0.000
M3	$\beta_1 = 1$	-0.008	0.019	0.000	-0.045	0.069	0.007	0.000	0.018	0.000	-0.003	0.012	0.000
	$\beta_2 = 0.5$	-0.004	0.035	0.001	-0.021	0.059	0.004	0.000	0.034	0.001	-0.002	0.022	0.001
							The Model by (1)						
M1	$\beta_1 = 1$	-0.102	0.021	0.011	-0.139	0.110	0.031	-0.088	0.020	0.008	-0.013	0.015	0.000
	$\beta_2 = 0.5$	-0.051	0.037	0.004	-0.066	0.077	0.010	-0.044	0.040	0.004	-0.007	0.027	0.001
M2	$\beta_1 = 1$	-0.077	0.020	0.006	-0.057	0.042	0.005	-0.078	0.020	0.006	-0.004	0.012	0.000
	$\beta_2 = 0.5$	-0.038	0.038	0.003	-0.028	0.053	0.004	-0.040	0.039	0.003	-0.001	0.022	0.000
M3	$\beta_1 = 1$	-0.101	0.021	0.011	-0.127	0.094	0.025	-0.080	0.021	0.007	-0.003	0.012	0.000
	$\beta_2 = 0.5$	-0.050	0.037	0.004	-0.061	0.067	0.008	-0.040	0.041	0.003	-0.002	0.022	0.001

Table S.3: The results of PH tests for each covariate using PCR data from all counties

Term for test	chisq	df	p_value
Serum PSA	303.671	1	<0.001
Age at diagnosis	41.548	1	<0.001
Insurance	110.116	1	<0.001
Appalachian	0.539	1	0.463
Radiation only vs. neither	77.051	1	<0.001
Surgery only vs. neither	60.864	1	<0.001
Surgery + Radiation vs. neither	32.973	1	<0.001
Treatment unknown vs. neither	0.529	1	0.467
Black vs. White	3.811	1	0.049
Others/Unknown vs. White	0.063	1	0.802
More aggressive stage vs. less	191.702	1	<0.001
Unknown stage vs. less	0.012	1	0.912

Figure S.1: The KM curves by county

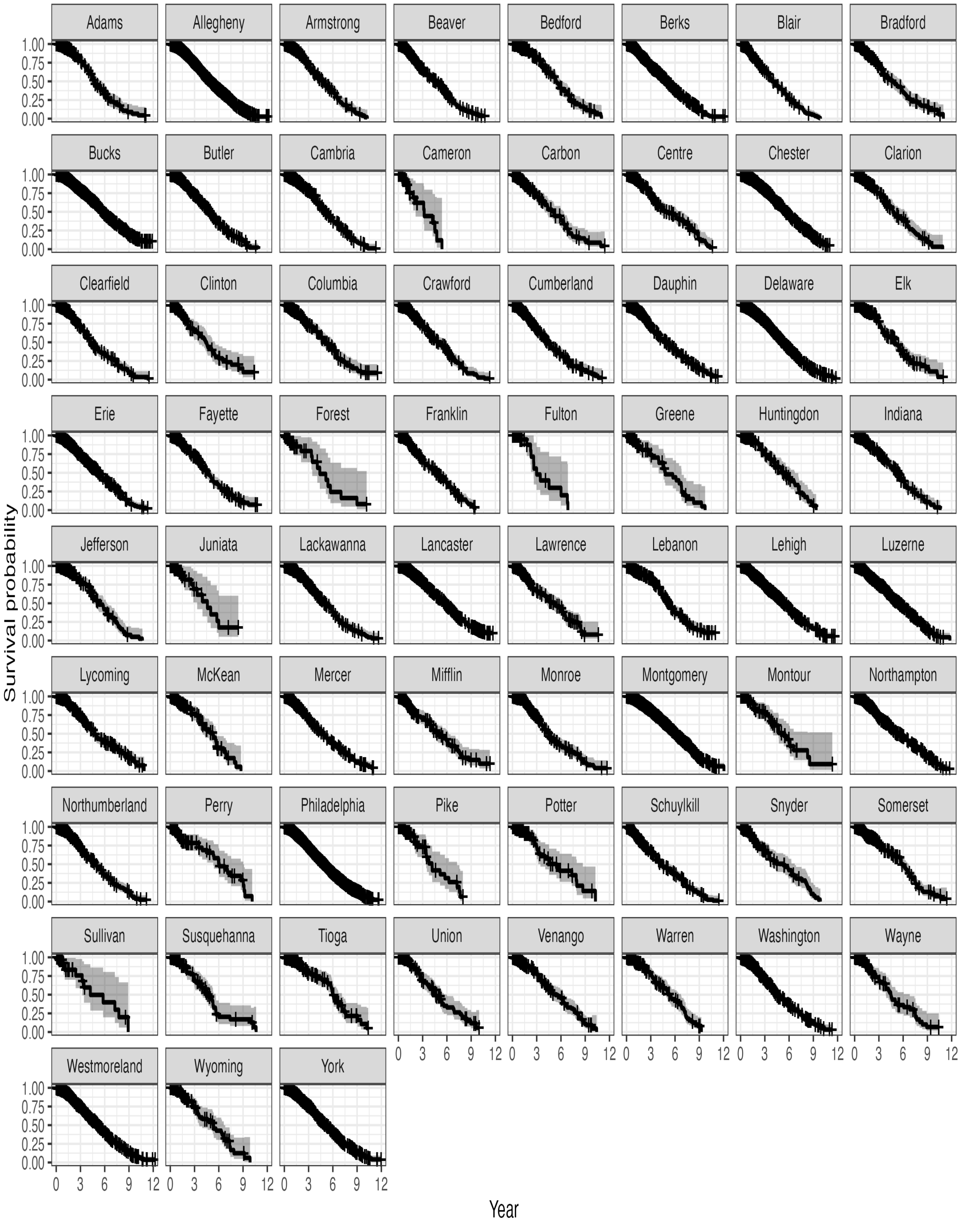

Figure S.2: The KM curve and hazard function for the PCR

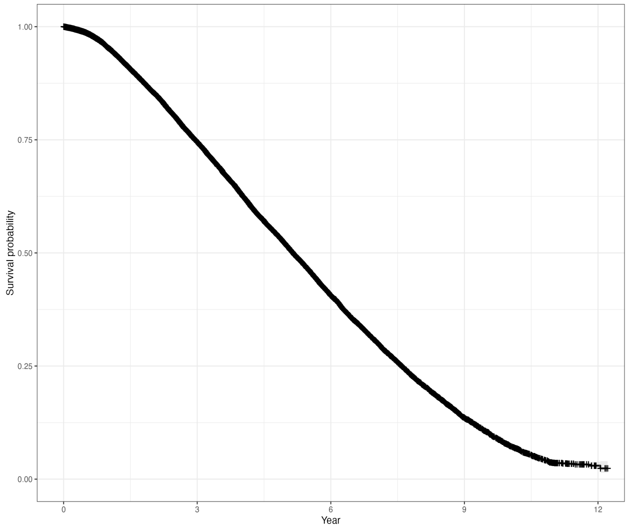

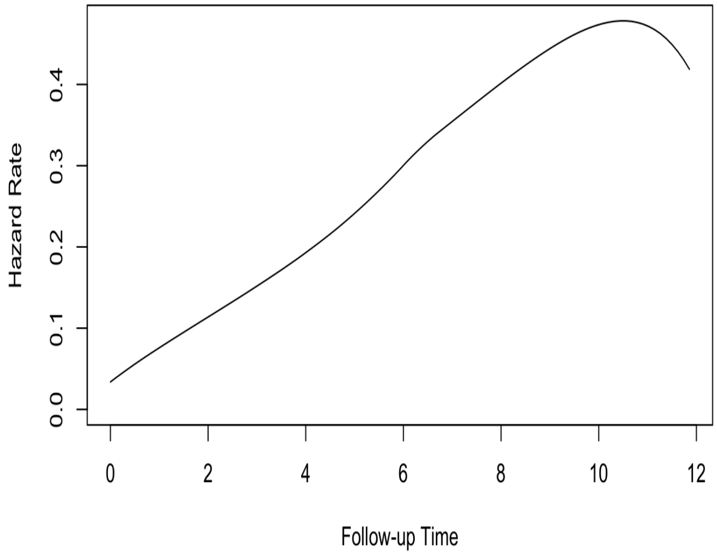

Table S.4: The sensitivity analysis using M3 (log-logistic) with different priors for PCR data analysis. IG: inverse Gamma; Diag: diagnoal metrix; EST: parameter estimate; CL: credible limit

Covariates	$\sigma^2 \sim IG(0.001, 0.001)$ $\mathbf{R} = Diag\{100, \ldots, 100\}$	$\sigma^2 \sim IG(0.1, 0.1)$ $\mathbf{R} = Diag\{100, \ldots, 100\}$	$\sigma^2 \sim IG(.001, .001)$ $\mathbf{R} = Diag\{10, \ldots, 10\}$	$\sigma^2 \sim IG(0.1, 0.1)$ $\mathbf{R} = Diag\{10, \ldots, 10\}$
	EST (95%CL)	EST (95%CL)	EST (95%CL)	EST (95%CL)
**Serum PSA**	-0.203 (-0.236, -0.167)	-0.228 (-0.229, -0.196)	-0.229 (-0.232, -0.190)	-0.227 (-0.226, -0.202)
**Age at diagnosis**				
>=65	REF			
<65	0.450 (0.401, 0.489)	0.476 (0.478, 0.534)	0.474 (0.472, 0.543)	0.487 (0.485, 0.544)
**Insurance**				
No	-0.167 (-0.235, -0.069)	-0.252 (-0.265, -0.078)	-0.151 (-0.142, -0.030)	-0.194 (-0.187, -0.052)
Yes	REF			
**Appalachian**				
No	REF			
Yes	-0.107 (-0.165, -0.081)	0.758 (0.713, 0.803)	3.671 (4.003, 6.009)	2.734 (2.853, 4.479)
**Treatment at diagnosis**				
Neither Surgery or Radiation	REF			
Surgery only	1.106 (1.054, 1.168)	1.137 (1.131, 1.219)	1.152 (1.129, 1.237)	1.166 (1.163, 1.253)
Radiation only	0.571 (0.542, 0.614)	0.584 (0.578, 0.671)	0.607 (0.610, 0.687)	0.603 (0.597, 0.667)
Surgery and Radiation	1.240 (1.081, 1.399)	1.283 (1.300, 1.450)	1.328 (1.307, 1.512)	1.320 (1.297, 1.481)
Others/Unknown	-0.224 (-0.448, -0.057)	-0.273 (-0.273, -0.064)	-0.294 (-0.281, -0.129)	-0.291 (-0.286, -0.129)
**Race**				
White	REF			
Black	-0.184 (-0.234, -0.114)	0.169 (0.141, 0.195)	-0.150 (-0.140, -0.061)	-0.163 (-0.168, -0.077)
Others/Unknown	0.665 (0.563, 0.784)	-0.422 (-0.521, -0.319)	0.712 (0.710, 0.868)	0.745 (0.748, 0.910)
**Stage of Aggressiveness**				
Less Aggressive	REF			
More Aggressive	-0.525 (-0.553, -0.486)	-0.577 (-0.574, -0.532)	-0.593 (-0.594, -0.544)	-0.584 (-0.584, -0.534)
Unknown	-0.072 (-0.136, -0.017)	-0.136 (-0.131, -0.063)	-0.155 (-0.138, -0.071)	-0.167 (-0.161, -0.108)
**Year at diganosis**				
Year=2004	REF			
Year=2005	-0.036 (-0.107, 0.078)	-0.625 (-0.660, -0.058)	-1.034 (-1.076, -0.099)	-0.668 (-0.684, -0.084)
Year=2006	-0.114 (-0.157, 0.022)	-0.129 (-0.197, 0.222)	-0.374 (-0.419, -0.090)	0.192 (0.080, 0.725)
Year=2007	-0.112 (-0.165, 0.037)	-1.267 (-1.435, -0.315)	-1.091 (-1.155, -0.361)	-1.175 (-1.373, -0.413)
Year=2008	-0.103 (-0.165, 0.050)	-1.581 (-1.771, -0.544)	-1.706 (-1.782, -0.620)	-1.733 (-1.897, -0.663)
Year=2009	-0.053 (-0.159, 0.110)	-0.148 (-0.089, 0.164)	0.453 (0.474, 0.776)	0.861 (0.831, 1.608)
Year=2010	0.052 (-0.030, 0.191)	0.446 (0.499, 0.727)	0.977 (0.993, 1.244)	0.599 (0.573, 0.826)
Year=2011	0.277 (0.193, 0.407)	1.364 (1.207, 2.622)	2.028 (1.653, 3.679)	2.268 (2.340, 3.848)
Year=2012	0.267 (0.193, 0.403)	1.391 (1.244, 2.552)	1.997 (1.653, 3.633)	2.290 (2.398, 3.802)
Year=2013	0.619 (0.536, 0.765)	2.038 (2.087, 2.924)	2.470 (2.242, 3.888)	2.119 (2.159, 3.000)
Year=2014	1.271 (1.018, 1.553)	5.443 (5.656, 8.339)	5.167 (5.337, 7.368)	5.439 (5.739, 8.000)
DIC	34867.03	21440.53	16397.44	16597.06

Figure S.3: The goodness-of-fit check based on Cox-Snell residuals. The left one is for the AFT model without considering the spatial-temporal dependency; the right one is for the AFT model M3 with log-logistic distribution and the priors of $\sigma^2 \sim IG(0.001, 0.001)$ and $\mathbf{R} = Diag\{10, \ldots, 10\}$ (the selected best candidate model in terms of the lowest DIC)

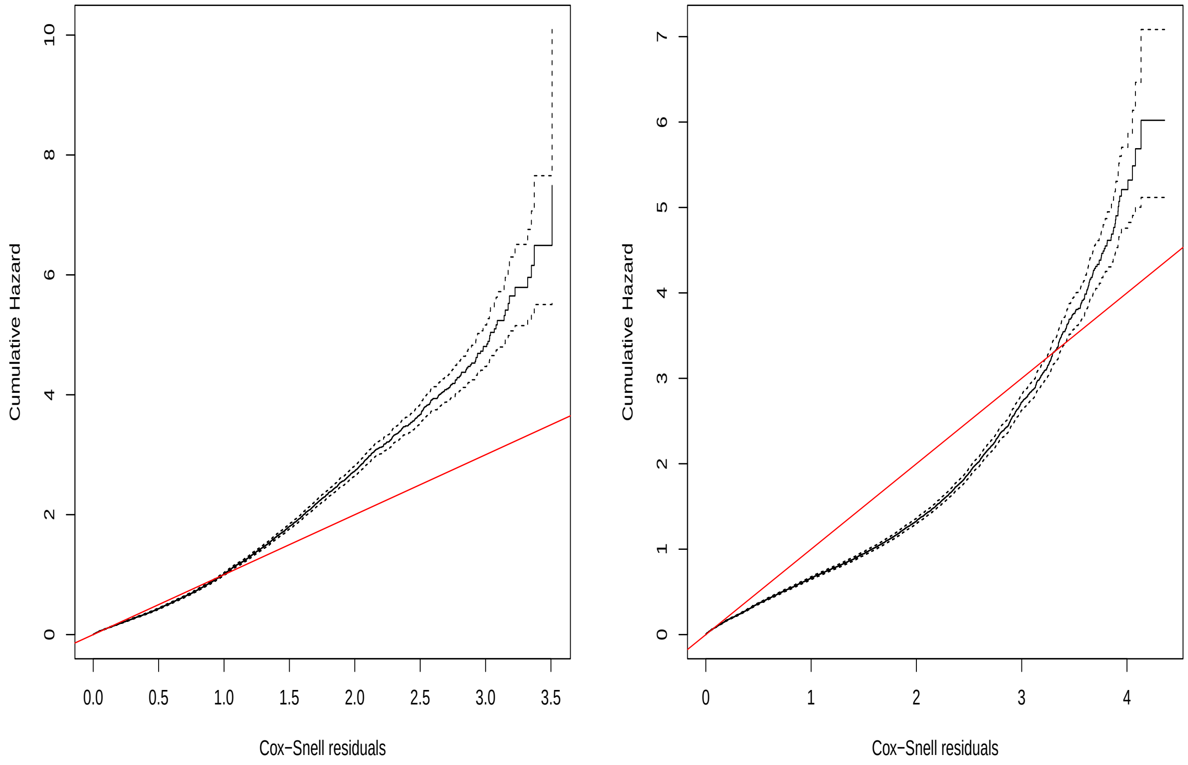

Supplement: Supplementary file 1 — Supplementary Material 1. [file 12874_2024_2201_MOESM1_ESM.pdf]
